# Uncovering Dangerous Cheats: How Do Avian Hosts Recognize Adult Brood Parasites?

**DOI:** 10.1371/journal.pone.0037445

**Published:** 2012-05-18

**Authors:** Alfréd Trnka, Pavol Prokop, Tomáš Grim

**Affiliations:** 1 Department of Biology, University of Trnava, Trnava, Slovakia; 2 Institute of Zoology, Slovak Academy of Sciences, Bratislava, Slovakia; 3 Department of Zoology and Laboratory of Ornithology, Palacký University, Olomouc, Czech Republic; University of Manitoba, Canada

## Abstract

**Background:**

Co-evolutionary struggles between dangerous enemies (e.g., brood parasites) and their victims (hosts) lead to the emergence of sophisticated adaptations and counter-adaptations. Salient host tricks to reduce parasitism costs include, as front line defence, adult enemy discrimination. In contrast to the well studied egg stage, investigations addressing the specific cues for adult enemy recognition are rare. Previous studies have suggested barred underparts and yellow eyes may provide cues for the recognition of cuckoos *Cuculus canorus* by their hosts; however, no study to date has examined the role of the two cues simultaneously under a consistent experimental paradigm.

**Methodology/Principal Findings:**

We modify and extend previous work using a novel experimental approach – custom-made dummies with various combinations of hypothesized recognition cues. The salient recognition cue turned out to be the yellow eye. Barred underparts, the only trait examined previously, had a statistically significant but small effect on host aggression highlighting the importance of effect size vs. statistical significance.

**Conclusion:**

Relative importance of eye vs. underpart phenotypes may reflect ecological context of host-parasite interaction: yellow eyes are conspicuous from the typical direction of host arrival (from above), whereas barred underparts are poorly visible (being visually blocked by the upper part of the cuckoo's body). This visual constraint may reduce usefulness of barred underparts as a reliable recognition cue under a typical situation near host nests. We propose a novel hypothesis that recognition cues for enemy detection can vary in a context-dependent manner (e.g., depending on whether the enemy is approached from below or from above). Further we suggest a particular cue can trigger fear reactions (escape) in some hosts/populations whereas the same cue can trigger aggression (attack) in other hosts/populations depending on presence/absence of dangerous enemies that are phenotypically similar to brood parasites and costs and benefits associated with particular host responses.

## Introduction

The evolutionary battle between dangerous enemies and their victims is one of the most exciting and most studied aspects of interspecific interactions [1]. In the last few decades, interspecific brood parasites, e.g., common cuckoos *Cuculus canorus* (hereafter: cuckoo) and their hosts became the focus of studies of antagonistic co-evolution and arms-races [2] with many exciting recent developments and discoveries [3], [4].

Due to the typically extreme fitness costs of acceptance of interspecific parasites [5], hosts have evolved multiple lines of defence. Host defences include recognition of and aggression against adult parasites (adult enemy discrimination; reviewed in [6]), recognition and destruction of parasite eggs before they hatch (egg discrimination; reviewed in [2]) and desertion or direct killing of foreign nestlings (chick discrimination; reviewed in [7]).

Later lines of defence are less beneficial than earlier implemented defences (see the “rarer enemy” hypothesis; [7]); therefore, it is surprising that nest defence, as a “front line” defence, is much less studied than egg discrimination [8]–[11]. Egg discrimination may not serve as an effective defence against parasitism especially in hosts that are victimized by parasites laying highly mimetic eggs and/or whose hatchlings evict host progeny [12]. In contrast, deterrence of laying parasites can more effectively reduce the host's likelihood of being parasitized [8].

Several previous studies have shown responses of hosts to brood parasites differ from those to other (non)threatening intruders, suggesting hosts can recognize brood parasites as special enemies [6], [13]–[19]. However, only a few studies have examined what specific cues hosts use to recognize them [20]–[22].

Currently, we know very little about salient cues that trigger specific host aggression against adult parasites. In this respect, the studies of host anti-adult parasite responses are lagging behind the studies of host responses to eggs. Egg discrimination studies have shown hosts recognize specific cues such as maculation [23], background colour [24] or their combination [25] and pay attention to cues located only at specific parts of the egg (the blunt egg pole) and ignore cues at other egg regions (the sharp egg pole; [26]). To parallel these advances in the study of egg discrimination, we have introduced a novel experimental approach to the study of adult enemy discrimination to find where the discrimination cues are located (i.e., front or rear body part?) and the identity of those cues.

Previous studies have suggested relevant cues for cuckoo recognition might be located on the head of the cuckoo (the yellow eyes, [27], [28]) or on the bottom part of its body (the barred underparts, [21], [22]). Cuckoo-hawk mimicry hypothesis suggests that the barred underparts of adult cuckoos facilitate brood parasitism (birds can mistake cuckoos for hawks and avoid attacking them [21], [22]). In contrast, another study [16] speculated, based on comparative evidence, that yellow eyes are an unlikely recognition cue; however, previous studies have tested only effects of head [27] or only effects of underparts [22]. Therefore, to understand the relative importance of these two stimuli both should be manipulated at once within one study.

**Figure 1:**
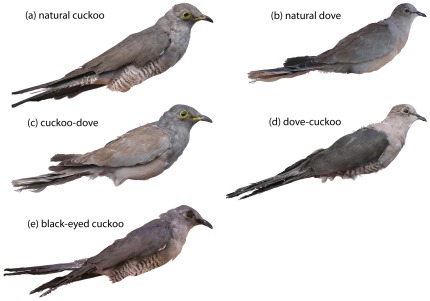
Dummies used in experiments showed all combinations of two hypothesized recognition cues (yellow eyes, barred underparts). Presence (+) and absence (−) of hypothesized recognition cues (yellow eyes/barred underparts) on particular dummies: (a) natural cuckoo (+/+), (b) natural dove (−/−), (c) cuckoo-dove (+/−), (d) dove-cuckoo (−/+), (e) black-eyed cuckoo (−/+). Dummies (c–e) were custom-designed for this study. All dummies were natural stuffed dummies (i.e., not artificially painted) in a life-like perching position similar to positions showed by cuckoos visiting host nests. Legs of dummies are not shown in this figure to save space and for visual simplicity.

Multiple dummies varying in the presence of both hypothesized recognition cues, i.e., yellow eyes and barred underparts, and all their combinations were used in this study (Fig. 1a–e). We tested (a) whether both or individual traits triggered specific recognition of cuckoos and (b) their relative importance (effect sizes). Further, to test a stimulus summation hypothesis (i.e., a stimulus is only effective or more effective when accompanied by another stimulus; [29]) we also tested (c) an interaction between the two potential recognition cues in our statistical models.

We selected great reed warblers *Acrocephalus arundinaceus* (hereafter: warbler) as a suitable model host species. This warbler is one of the most widespread cuckoo hosts (e.g., [30], [31]), having evolved an advanced ability to reject foreign eggs [26] and shows strong aggression against adult cuckoos [31], [32]. Here, following heuristically strong paired experimental design, we simultaneously presented dyads of taxidermic mounts at host nests [33], [34]. We addressed three hierarchical questions: (a) Do warblers recognize cuckoos as a special threat? (b) In what body region (front or rear) of the cuckoo are recognition cues located? (c) Is the bright yellow eye a specific cue for cuckoo recognition?

## Methods

### General field procedures

The study was performed in a fishpond system near Štúrovo (47°51′N, 18°36′E, 115 m a.s.l.), south-western Slovakia in 2011. Great reed warbler populations (40 to 60 pairs) nest in narrow strips of the reeds bordering the ponds.

After the arrival of warblers from African wintering grounds (from mid-April till mid-May), we mist-netted adult birds and individually banded them with aluminium rings and unique combinations of colour rings. During breeding season (from mid-May till mid-July) we systematically searched for warbler nests in the same areas. Nests were checked daily to individually mark each host egg in the laying order and to detect cuckoo parasitism. As each host egg was marked soon after being laid and local warblers typically eject natural cuckoo eggs only after several days (unpubl. data), it is unlikely we mistakenly assigned parasitism status to any nests.

Our research followed guidelines of the Animal Behavior Society for the ethical use of animals in research. Licenses and permission to ring and handle the birds were provided by the Ministry of Environment of the Slovak Republic, No. 269/132/05-5.1pil and No. 7230/2008-2.1pil.

### Taxidermic mounts

We used taxidermy mounts of a cuckoo, a control, and their body part combinations. Previous studies have used the pigeon *Columba livia f. domestica* [18], [35]) or collared dove *Streptopelia decaocto* [21], [30]) as control dummies to test whether hosts discriminate brood parasites as special enemies. We decided to use the latter innocuous species dummy, as the collared dove (hereafter: dove) is similar to the cuckoo in size, shape, plumage and flight, and is familiar to warblers at our study site (cf. [16]). Warblers from another population were shown to still be able to differentiate between the two [30]. Using the pigeon which is even more similar to the cuckoo than the dove would increase risks that hosts would commit too many recognition errors as shown in several previous studies [16], [35]–[37].

All specimens were stuffed in a similar posture (which might otherwise affect host responses, discussed in [22]). The mounts were in life-like positions with folded wings and their heads pointing forwards. We employed two different specimens of each dummy type o reduce the possibility that differences between treatments could be caused by a particular specimen [6]. The particular specimen was chosen randomly for each experiment. Similar to previous studies we did not reveal any differences in host responses to different replicate dummies of the same type [16], [17], [22]. To keep mounts in good condition and to keep their appearance similar across tested nests, experienced taxidermist (AT) preened the mounts before each experiment.

### Experimental procedures

We used paired experimental design employed by Ligon and Hill [33]. We used the paired approach because successive presentations of similar-looking intruders (e.g., cuckoo and dove in the present study) increases the risk of reinforcement or habituation [6], [38]. This may be a serious problem especially in highly aggressive hosts, like warblers (discussed in [34]). Within each experiment (see below) a dyad consisting of two different dummies was simultaneously presented to hosts. To avoid the risk that nest owners would see the dummies before the start of the experiment, we arranged the dummies near the nest when the nest owners were not present at the nest or its vicinity. We placed the mounts 0.5 m from the focal nest at the same height above water level, facing the nest rim, and 0.8 m apart from each other. We randomized the side where each mount was presented (i.e., left or right from observers' direction). The reeds around each nest were arranged in order to provide the nest owners with a good view of both mounts at the same time. We did not accompany dummy presentations with playback calls (as in [30]) because cuckoos do not call when visiting host nests [39].

Observations (all by AT and PP) were made from a blind (placed ∼5 m from the focal nest) that afforded the best views of both mounts. One observer recorded warbler responses to the left-hand side mount whereas the other observer recorded responses to the right-hand side mount. The position of observers was randomized across nests. Most of the trials were also videotaped with a digital video camera (Sony DCR-HC17E) to validate the field notes.

Pilot experiments in 2010 showed warblers decrease their responses after ca 1 min from their first attack. To avoid this habituation in 2011 experiments (this study) we set the length of experiment to 1 min. Each experiment began at the moment of the first contact-attack by one of the nest owners at any of the two dummies. At all 54 tested nests at least one of the dummies within a dyad was attacked, typically immediately after the arrival of nest owners (therefore we did not analyse lag between arrival and first contact attack in this study). Host responses were recorded as the number of contact attacks per 1 min and the experiment was stopped to avoid dummy destruction [32] and host habituation ([6]; pers. obs. during pilot experiments).

We used only the rates of contact attacks to evaluate host behaviour, although avian responses to intruders near the nest also include other activities, such as calling, dive flights, or distraction displays [6]. However, as we wanted to test whether hosts discriminate between two *simultaneously* presented dummies, many of these activities, including latency of arrival, calling, etc., could not be assigned to a particular dummy within the dyad. Moreover, physical attacks may be more effective than alarm calling in driving cuckoos from hosts nests, at least in larger body sized hosts like the warbler [39]–[41].

### Experiment 1: “Do hosts recognize the cuckoo as a special enemy?”

The ability to recognize specific enemies may vary intraspecifically: some host populations, e.g., those frequently parasitized, differentiate between brood parasites and innocuous species; whereas, other populations, e.g., those less parasitized or non-parasitized, do not [42]. For example, warblers in an area of extremely high parasitism rate (∼65%, [30]) recognize the cuckoo as a special threat [30]. Our study warbler population was parasitized less frequently (∼30%) and in a similarly parasitized population (36%, [32]) warblers did not recognize cuckoos [37]. It would have been premature to manipulate specific potential recognition cues without establishing first that hosts do indeed recognize the adult cuckoos specifically (see also [20]); therefore, Experiment 1 was used to test whether our study population showed specific enemy discrimination ability. We tested the “specific enemy recognition” hypothesis by simultaneous presentation of dummy dyad consisting of a dangerous brood parasite (natural cuckoo; Fig. 1a) and an innocuous intruder (natural dove; Fig. 1b) as a control. We predicted more aggressive host responses to cuckoo than to dove dummies.

### Experiment 2: “In what body region are recognition cues located?”

Egg discrimination studies have shown egg recognition cues might not be distributed across the whole egg surface but may be located only in specific parts of the egg phenotypes. Manipulation of either sharp or blunt egg pole of host eggs showed hosts rely on cues located on the blunt egg pole only [26], [43]. This experimental design has not been used in studies of adult enemy recognition so far. We employed custom-made dummies where a cuckoo front body part and a dove rear body part were combined (cuckoo-dove; Fig. 1c) or vice versa (dove-cuckoo; Fig. 1d). We tested the “body region hypothesis” by simultaneous presentation of cuckoo-dove plus dove-cuckoo dummy dyad. We predicted warblers would respond to the cuckoo-dove dummy more strongly (similar to the natural cuckoo dummy response) because the front body part is typically the target of contact attacks from birds defending their nests [16], [27], including the warbler [34].

### Experiment 3: “What is the specific cue for enemy recognition?”

Eye colour was suspected to be a specific recognition cue [27] based on the fact that the cuckoo yellow iris and eye-ring are both conspicuous and not shared by other species sympatric with warblers in our study area (pers. obs.; see also Discussion). Sparrowhawks *Accipiter nisus* also show yellow eyes; however, *Acrocephalus* warblers were never recorded in the diet of sparrowhawks in Central Europe where this study was performed (*n* = 85 256 prey items, pp. 439–441 in [44]). We never observed sparrowhawks in our study site during the breeding season (pers. obs.) in contrast to other sites (England) where sparrowhawks are known to be in contact with *Acrocephalus* warblers and do prey on them (N. B. Davies, pers. comm.). AT prepared a “black-eyed cuckoo” dummy with eye (iris plus eye-ring) colour being covered by black tempera colour (Fig. 1e). We tested the “eye colour as the specific recognition cue” hypothesis by simultaneous presentation of natural cuckoo (the same *type* of dummy as in Experiment 1 but in Experiment 3 we used different specimens) and black-eyed cuckoo dummy dyad. We predicted hosts would respond more strongly to the natural cuckoo dummy and responses to the black-eyed dummy would be low and similar to dummies that should be perceived as innocuous by hosts (dove, dove-cuckoo) if the major recognition cue was the eye colour.

### Confounding factors and randomization

Previous research identified many variables that could affect host responses to brood parasites [45], such as timing in the breeding season [46], nesting stage [47], daytime [39], nest reproductive value [18], mating status [48], prior recent experience with parasites near the nest [38], and host sex [32]. In the present study we therefore specifically aimed at avoiding these potential confounding factors. Each warbler pair was tested only once and all experiments were conducted only on the first unpredated and non-parasitized monogamous nests. All dummy presentations were made consistently on the first day of the incubation period (i.e., the first day when a new egg did not appear in the nest after previous daily egg laying) and between 7:00 and 11:00 (CET). Similarly, we successfully randomized the presentations of dummy dyads throughout the breeding season (i.e., both range and average presentation dates were statistically identical across Experiments 1–3, average dates: ANOVA: *F*_2,51_ = 0.17, *p* = 0.85).

Moreover, we statistically controlled for other potentially relevant confounders (date in the season, clutch size, sex) and direction of host pair arrival that also might influence their target. More specifically, when hosts were arriving to the nest from a direction that did not allow them to see both dummies roughly at the same time, they could respond more strongly to the dummy they spotted first. Therefore, we included in GLMM (see below) a variable “host arrival direction” (for the first arriving pair member) with following level coding: 0 = first member of host arrived “directly” to the nest and had a chance to see both dummies at the same moment, 1 = dummy side, when host first saw the focal dummy and −1 = opposite dummy side, when host first saw the dummy paired with the focal dummy. We considered only arrival of the first member of the pair because the first member always started to respond before the arrival of the second pair member.

### Statistical analyses

We Box-Cox transformed the response (number of contact attacks +0.1), thus, all models (i.e., full, partially reduced, and final minimum adequate models) showed normal distribution of residual errors (Shapiro-Wilk tests, all *p* = 0.22–0.42). We first built the full general linear mixed model (GLMM, normal error distribution, parameters estimated by REML, degrees of freedom calculated using Kenward-Roger method) with pair id as a nominal random effect, transformed number of contact attacks as continuous response and following predictors: dummy type (nominal), date in the season (continuous), including its quadratic term (to check for possible non-linear seasonal trends), clutch size (continuous), host arrival direction (nominal), first attacking sex (nominal).

We did not enter a potential variable “type of experiment” (Experiment 1–3), because the paired nature of the experiment was already modelled by including pair id as a random effect. Due to the timing of experiments (1^st^ day of incubation) we did not include first egg laying date (as in some other studies) into our models because that variable is highly correlated with experimental date, consequently causing a statistical problem of multicollinearity [49]. Post-hoc comparisons between all dummies were based on Tukey-Kramer HSD test with *α* = 0.05 (conclusions remained the same when *α* was varied between 0.10 and 0.001).

Some previous studies of nest defence assessed bird behaviour on a categorical scale (contact attack: yes/no; [15], [32], [50]). To test how behavioural coding may affect results (which is highly relevant e.g. for future meta-analyses), we re-coded our original continuous data on such a categorical scale and analysed both categorical and continuous data with non-parametric Wilcoxon sign-rank tests.

In a separate analysis we examined what specific cues are used by hosts to recognize the cuckoo. We again used GLMM as described above but substituted “dummy type” with predictors “eye” (eye colour of particular dummy yellow = 1, or not = 0) and “underparts” (underparts of particular dummy barred = 1, or plain without barring = 0). We also included an interactive term (eye×underparts) to test the stimulus summation hypothesis ([29], see Introduction).

Test statistics and *p*-values reported in Results for non-significant terms are from a sequential backward elimination procedure just before the particular term (being the least significant) was removed from the model. The final minimum adequate model contained only significant predictors. Although we had specific *a priori* directional predictions the use of one-tailed tests in ecological studies is inappropriate [51]; therefore, all tests in the present study are two-tailed. All analyses were performed in JMP 8.0.1. (SAS Institute Inc., Cary, NC, USA).

## Results

**Figure 2:**
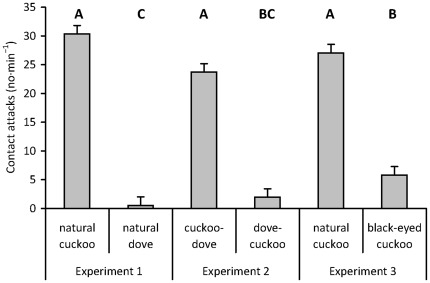
Great reed warbler aggressive responses (least square means + s.e.) to experimental dummies (**Fig. 1**). Results from GLMM with Box-Cox transformed responses and normal residual errors (Table 2). Least square means from the final model were back-transformed to original scale (number of contact attacks/1 min) for easy interpretation. Different letters indicate statistical differences between groups according to GLMM (Tukey HSD, *p*<0.05).

In Experiment 1, all tested pairs (*n* = 17) consistently responded more strongly to the natural cuckoo dummy than to the natural dove dummy resulting in large effect sizes (Fig. 2, Table 1). In fact, all pairs attacked the cuckoo whereas 71% of the same host pairs ignored the dove completely (i.e., did not make a single contact attack).

**Table 1:** Paired comparisons of great reed warbler responses to simultaneously presented dummies within experimental dyads (see Methods and Fig. 1).

Experiment	*N* (host pairs)	Continuous response		Categorical response	
		Z	*p*	Z	*p*
Natural cuckoo vs. natural dove	17	−76.5	<0.0001	−39.0	0.0005
Cuckoo-dove vs. dove-cuckoo	19	−94.0	<0.0001	−7.5	0.06
Natural cuckoo vs black-eyed cuckoo	18	−75.5	0.0003	−5.0	0.13

Responses were measured either as number of contact attacks (Continuous response) or re-coded as presence vs. absence of attacks (Categorical response). See Discussion for rationale behind and implications of categorical re-coding. Differences tested with Wilcoxon sing-rank tests.

In Experiment 2 all tested pairs (*n* = 19), with the exception of one, reacted more strongly to cuckoo-dove than to dove-cuckoo (Table 1) with responses to cuckoo-dove being statistically identical to responses to natural cuckoo (Fig. 2). All cuckoo-doves were attacked while 26% of host pairs ignored the dove-cuckoo completely.

Finally, all tested pairs (*n* = 18), with the exception of two, were more aggressive towards natural cuckoo than to black-eyed cuckoo (Experiment 3; Table 1). Similarly to Experiment 1, all natural cuckoos were attacked. In contrast, 22% of host pairs from Experiment 3 ignored the black-eyed cuckoo completely. Responses to black-eyed cuckoo were similar to responses to dove-cuckoo (Experiment 2; Fig. 2) but slightly larger than those to dove alone (Experiment 1).

Importantly, removal of barred underparts *per se* had negligible and a statistically non-significant effect on host responses (compare natural cuckoo from Experiments 1 and 3 with cuckoo-dove from Experiment 2; Fig. 2). In a striking contrast, removal of yellow eyes *per se* dramatically decreased host aggression against the dummy (compare natural cuckoo from Experiments 1 and 3 with black-eyed cuckoo from Experiment 3; Fig. 2).

Based on re-coded data on a categorical scale we found that Experiment 1 still showed significant differences in warbler responses between dummies within a dyad. However, Experiment 2 was marginally non-significant and Experiment 3 failed to detect the differences (Table 1; see Discussion for methodological implications of this result).

No potential confounding variables affected host responses (Table 2). Only the dummy type showed a significant effect on host aggression independent of other factors. Importantly, the random effect (pair id) was negligible and non-significant in all models (likelihood ratio tests), i.e., warbler pairs did not vary in the magnitude of the difference in their responses between dummies within a dyad.

**Table 2:** Great reed warbler responses to multiple dummy types.

Minimal adequate model	df	*F*	*P*
Dummy type	5, 75.77	43.74	<0.0001

Removed predictors			
Experimental date – linear	1, 50	0.04	0.84
Experimental date – quadratic	1, 49	1.25	0.27
Final clutch size	1, 48	1.02	0.32
Host arrival direction	2, 68.29	0.27	0.76
First attacking sex	2, 46	0.76	0.48

For photographs of dummies see Fig. 1, for effect sizes see Fig. 2. Results from GLMM. For explanation of variables and analyses see Methods.

In most experiments (76%, *n* = 54 nests) females launched the attack. Males rarely (7%) started to attack before females, in the remaining cases (17%) females and males launched the attack simultaneously. However, the sex of the first attacker had no statistically detectable effect on overall aggression (Table 2).

**Figure 3:**
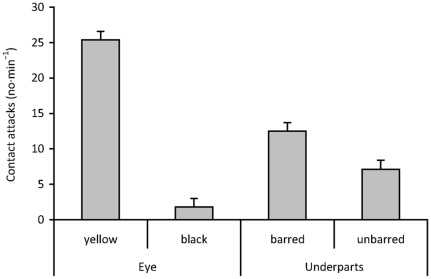
Great reed warbler aggressive responses (least square means + s.e.) to specific potential recognition cues (eye colour, underpart barring). Results from GLMM with Box-Cox transformed responses and normal residual errors (Table 3). Least square means from the final model were back-transformed to original scale (number of contact attacks/1 min) for easy interpretation.

We found no evidence for stimulus summation – the interaction between colour of eye and underparts was non-significant (Table 3). Both eye and underparts colour were significant but eye colour showed a much larger effect size than underparts colour (Fig. 3). Specifically, presence of barring increased aggression ∼1.8-times, whereas presence of yellow eyes increased aggression ∼14.1-times compared to absence of the two traits, respectively (Fig. 3). Both traits together explained 66% of variation in host aggression. Partitioning of variance (according to Zuur et al. [52], p. 75–77) showed that 63% were attributable to pure effect of eye colour and 3% were attributable to pure effect of underparts (variance shared by the two variables was negligible).

**Table 3:** Effects of specific recognition cues on great reed warbler aggression near the nest.

Minimal adequate model	df	*F*	*p*
Eyes	1, 51.81	181.98	<0.0001
Underparts	1, 87.16	9.65	0.003

Removed predictors			
Eyes×Underparts	1, 80.68	2.48	0.12
Experimental date – linear	1, 49.06	0.01	0.92
Experimental date – quadratic	1, 47.99	1.31	0.26
Final clutch size	1, 48.18	1.64	0.21
Host arrival direction	2, 68.15	0.18	0.83
First attacking sex	2, 45.91	0.50	0.61

Yellow eye colour and barred underparts (typical for natural cuckoos) were either present or absent on a particular dummy (Fig. 1). Statistics are from GLMM models with Box-Cox transformed response, for effect sizes see Fig. 3.

## Discussion

Differences and similarities in host responses to various dummy types revealed where and what the specific cue used by hosts to detect a dangerous enemy near their nests was. Great reed warblers in our study population (a) recognized the cuckoo as a special enemy (Experiment 1), (b) focused on a cue located in the front body region of the intruder (Experiment 2), and (c) relied mostly on a single recognition cue, i.e., the colour of intruder's iris and eye-ring (Experiment 3). Hosts showed weak and mostly similar (see effect sizes) responses to natural doves, dove-cuckoos and cuckoos with blackened eyes (Fig. 2). In a striking contrast, the same hosts showed consistently and dramatically higher levels of aggression to natural cuckoos and cuckoo-doves, respectively, and their responses were statistically the same between these two kinds of stuffed dummies (Fig. 2, Table 2). Additional analyses based on presence/absence of phenotypic traits of yellow eye and barred underparts confirmed that eye colour is the primary recognition cue (Fig. 3, Table 3). In fact, 95% of explained variation was attributable to eye colour only (minimum adequate model R^2^ = 0.66). However, to validate whether yellow eye colour is truly a critical cue for cuckoo recognition, additional eye colour manipulation experiments (e.g., the cuckoo with black eyes vs. dove with yellow eyes) are needed. Underpart barring provided a subsidiary cue for recognition of cuckoos as special enemies – the variable was statistically significant but the effect size was small.

### Intra- and inter-specific variation in host enemy discrimination

Interestingly, whereas the presence of barred underparts *decreases* mobbing by non-hosts and reed warblers [21], [22] as predicted by the “cuckoo-hawk mimicry” hypothesis, the aggression of great reed warblers was slightly *increased* in the presence of this cue (this study). However, this finding does not reject the hawk-mimicry hypothesis for the following reason. The differences between great reed warbler and reed warbler responses make sense when considering ecological context of the study populations and recoverability of costs of the host behaviour (the latter *sensu* [53]).

In the UK study site sparrowhawks are common (N. B. Davies, pers. comm.) and thus hosts benefit from *fearing* any bird with barred underparts. Such host response (not attacking or escaping from barred intruders) may increase the risk of cuckoo parasitism but hosts can reject already laid cuckoo eggs (i.e., costs are, at least partly, recoverable). In contrast, not fearing barred underparts may lead to a host's death – in the case when the barred intruder is the sparrowhawk (i.e., costs are not recoverable). In the Slovak study site (this work) sparrowhawks were not present during the breeding season (as the great reed warblers are migratory they are effectively allopatric with sparrowhawks at our study site). There was no other bird species with barred underparts present sympatrically with warblers in our study site; thus, the barred underparts uniquely denote the cuckoo in this particular area. In contrast to the UK, at the Slovakian site great reed warbler populations benefit from *attacking* any bird with barred underparts. Specifically, ignoring barred underparts has no benefits and only costs (i.e., cuckoo parasitism at non-defended nests). In contrast, attacking a bird with barred underparts may lead to benefits (in the case of successfully deterring the cuckoo from a host nest).

Although we cannot strongly conclude whether the differences between UK and Slovak sites are explained by this scenario (or stem from alternative factors) this discussion still leads to a novel and exciting hypothesis that might explain variation in host enemy discrimination both intra- and inter-specifically: “a particular cue can trigger fear reactions in some hosts/populations whereas the same cue can trigger aggression in other hosts/populations depending on presence or absence of dangerous enemies that are phenotypically similar to brood parasites and costs and benefits associated with particular host responses”. This hypothesis is in line with the theory that predicts Batesian mimicry to be less successful when the relative frequency of the mimic outnumbers the model [54]. This is also supported by empirical data from our study population: warblers mobbed sparrowhawks almost as much as cuckoos [34].

However, we stress that our results are not directly comparable with the previous studies [21], [22] because of different behavioural variables measured: contact attacks in the former whereas approach distance and vocalizations in the latter. Still, this has no bearing on the major messages of our study, where: (a) the primary enemy recognition cue seems to be eye colour with underpart appearance playing a secondary role and (b) effect sizes (Fig. 2,3) provide the critical information needed to assess the importance of a particular recognition cues in studies of discrimination behaviour in animals.

While attacks to the head could indicate this is where the recognition cue is located, it could also be because obscuring the vision of a cuckoo is likely to be a more effective deterrent than attacking its underparts. Although this hypothesis (recognition cue is different from the target of attacks) deserves testing in other hosts and populations, it is not supported in our study warbler population – the experiment with black-eyed cuckoo clearly indicates the yellow eye *per se* is the single most important cue triggering host aggression. This is because all other potential cues on the external phenotype of the intruder were identical between black-eyed cuckoo and natural cuckoo (Fig. 1). This excludes a possibility that hosts recognized the cuckoo as such based on a non-eye cue and then directed their attacks at intruder's eyes.

Both iris and eye-ring are bright yellow in cuckoos. However, we did not separately manipulate the two traits because it is unlikely that hosts would be able to differentiate them as two separate cues due to the very dense reed bed habitat with limited visual detection of details and very fast host responses. On the other hand, previous studies have shown great reed warblers are able to recognize cuckoos from sparrowhawks ([34]; see also [17]) despite the eye colour similarity between sparrowhawks and cuckoos. Therefore, additional traits, namely beak size/shape [20] and body posture [22] may play a role in enemy recognition ([27], p. 116). This could be tested in future studies.

### Recognition errors

Warblers showed very clear discrimination of the cuckoo from other simulated intruders. Still some pairs committed recognition errors by also attacking dummies that did not bear the critical recognition cues and even responding to the latter dummies more strongly than to dummies with the “correct” cue, although very rarely (1 out of 19 pairs in Experiment 2; 2 out of 18 pairs in Experiment 3).

These recognition errors might be explained by host age and/or experience (see also [38]). However, it is highly unlikely that host age affected conclusions of the present study due to (a) treatment randomization throughout the breeding season (date in the season is a surrogate measure of female age [38]), (b) absence of any clear age-related variation in warbler aggression found in another set of experiments in the same population (Trnka and Prokop unpubl. data), (c) 100% aggression in responses to natural cuckoo strongly supports the conclusion that host age does not affect probability of attack (it is highly unlikely that all tested females would be old, experienced and consequently aggressive and good discriminators with a sample size of 17 in Experiment 1). Alternatively, carry-over aggression between two dummies within a dyad could be partly responsible for apparent recognition errors [6]; however, if present, such an effect would be minimal – see overall clear and large differences in responses to two dummies within a dyad (Fig. 2).

Importantly, even species unsuitable as cuckoo hosts were documented to show some aggression toward cuckoos (e.g. [14], [15]). Some species are aggressive against any intruders near the nest, including innocuous ones [55]. We suspect this may hold true at the *intra*specific level: particular *individuals* may be aggressive against any strangers near the nest; thus, failing to show a clear enemy recognition (which may be typical for a *population* as a whole).

### Methodological and analytical aspects and recommendations

In the present study we specifically aimed at avoiding all potential confounding factors detected in previous studies by several means. First, we carefully randomized potential confounders including observer and dummy positions (see Methods). Second, we statistically controlled for some potentially relevant confounders including factors not considered in previous studies (e.g., direction of host pair arrival that might influence their target). Third, we excluded nests where other confounding factors might have played a role and where sample sizes per level of a confounding factor would result in highly unbalanced designs (e.g., female mating status in monogamous vs. polygynous pairs). We suggest future studies might benefit from utilizing these approaches.

Responses to natural cuckoo were statistically identical in Experiment 1 (natural cuckoo vs. natural dove) and Experiment 3 (natural cuckoo vs. black-eyed cuckoo). This finding suggests our sample sizes per treatment (*n* = 17–19 across experimental dyads) were sufficient to capture the biological reality of our study population and increasing sample sizes would not affect our parameter estimates and conclusions in other treatments. This conclusion is also supported by the large observed effect sizes (Fig. 2).

In a parallel analysis, we re-coded our original continuous data (no. of contact attacks) on a categorical scale (presence/absence of attacks), thus mimicking the methodological approach of some previous studies (e.g., [13]–[16]). Statistical tests based on categories failed to detect some statistically highly significant differences that were revealed by the test based on continuous response data, i.e., on original non-simplified observations of biological reality (Fig. 2). Thus, measuring host behaviour on a (a) continuous vs. (b) categorical scale can, in some particular data sets, lead to contradicting conclusions (continuous data: hosts do discriminate, categorical data: host do not discriminate). Thus, more caution in describing host behaviour may be beneficial for detecting existing discrimination abilities of hosts. We conclude the reduction of natural continuous variation in host responses into artificial categories, e.g., when the dummy is removed after the first host attack or due to binary coding of host responses [13]–[16], [32], [50] may be misleading and should be avoided in future studies (see also [34]).

The majority of “stuffed dummy” studies were based on the successive presentation of single mounts (e.g., [17], [10], [11], [15]) while other studies have employed simultaneous presentation of dummies (e.g., [33]). We adopted the latter approach because successive presentations of similarly-looking intruders (e.g., cuckoo and dove in the present study) increased the risks of reinforcement or habituation [6], [38]. This may be a serious problem especially in highly aggressive hosts, like great reed warblers. We are confident that the “simultaneous presentation of dummies” design is valid because our results and conclusions are in line with those from studies that used the alternative “successive presentation of dummies” design (discussed in [34]). Warbler responses were not likely affected by the presence of the second dummy because host reactions were similar to those to single mounts (cuckoo only, or dove only) in pilot experiments. Further, responses to natural cuckoo were statistically identical in experiments where the second dummy was either the natural dove (Fig. 1a) or black-eyed cuckoo (Fig. 1e). Finally, our novel conclusion that the major discrimination cue is located on the head of an intruder is supported by results of V. Bičík (unpubl. data) from *non*-simultaneous dummy presentations in another cuckoo host (see discussion of a “cue isolation experiment”, p. 172 in [28]).

### Can recognition cues be context-dependent?

Previous studies found some regular cuckoo hosts recognize cuckoos as special enemies [17], [18]. Further, they revealed both unsuitable [21] and suitable [22] hosts mistake cuckoos for sparrowhawks and the cue responsible for this host deception is the cuckoo's barred underparts. Our data both support and modify this conclusion. Another study showed that great reed warblers can distinguish cuckoos from sparrowhawks [34]. In the present study, we additionally showed warblers do recognize barred underparts as a recognition cue. Still, the barred underparts cue is not necessary to release host discrimination of cuckoos from innocuous enemies. This is because warblers frequently attacked “hybrid” cuckoo-dove dummies although these lacked the barred underparts (Fig. 1c) and mostly ignored dove-cuckoo dummies although these did show barred underparts (Fig. 1d). However, these differences do not support the “hawk-mimicry” hypothesis because host aggression was not reduced but increased by underparts barring (Fig. 2; for possible explanations see discussion above). This finding highlights the need for considering multiple candidate recognition cues in future studies of enemy discrimination.

This study suggests hosts may use the eye colour and underparts appearance to recognize the cuckoo as a special enemy near their nest. However, predation of clutches/broods, predation of adult birds and egg-laying by cuckoos are very quick phenomena [40], [56]; therefore, when recognizing an intruder near their nest, hosts need to act very fast as they do not have enough time for prolonged detailed assessment of intruder cues [34]. This suggests hosts base their enemy recognition on only few cues – ideally a single reliable conspicuous cue [33]. This may be especially important in dense reed-beds with limited visibility. Our finding of a major effect of eye colour and minor effect of underpart appearance makes sense in the light of this logic: the yellow eye is very conspicuous from the typical direction of host arrival (from above) and other species that could enter the vicinity of host nests do not show this trait (at least no birds that may be mistaken with the cuckoo due to their roughly similar body size and behaviour). In contrast, barred underparts are poorly/partly visible or even not visible at all because they are visually blocked by the upper part of the intruder's body (note warblers typically arrive high in reeds and the cuckoo is below, near the host nest). This visual constraint may reduce usefulness of barred underparts as a reliable recognition cue under a typical biologically relevant situation near host nests.

Importantly, any visual cue can be effective solely when the relevant signal receivers are able to see it. Barred cuckoo underparts could trigger passerine aggression during mobbing of flying or perched cuckoos (this species typically perches high in the canopy; thus, mobbers approach from *below*). In contrast, when cuckoos are encountered near hosts nests (typically found in low vegetation or on the ground, [39]) the nest owners typically arrive from *above* (pers. obs., photographs in [27]). Consequently, they have little chance to see underparts of the intruder. Thus, we speculate hosts may use recognition cues depending on the particular ecological context (see also [57]). Specifically they may rely on cues that are more likely to be visible in a particular situation: barred underparts when mobbing perched or flying cuckoos, yellow eyes when surprising cuckoos near their nests. While this “context-dependent recognition cues” hypothesis has been looked at in ants [58] and fish [59], it provides an exciting avenue for future enemy discrimination research in avian brood parasite-host systems.

In a general context of how animals recognize enemies our conclusions parallel findings from a diverse array of study systems. For example, natural eyes, eye spots and eye colour seem to be a recurrent theme of predator-prey interactions (e.g. [60]) and a classic text-book example of warning phenotypic traits [54]. Additionally, the direction of an intruder's gaze affects escape and, alternatively, attack reactions across a wide phylogenetic spectrum, from snakes [61], to birds [62] and mammals [63]. Further, yellow colour has been repeatedly shown as a stronger deterrence stimulus then some alternative colours (for a case study see [64], for review see [65]). Finally, eyes often appear to be specifically targeted in attacks, most likely because they can be physically fragile (and less expendable) compared to other body parts [54]. Future research will show whether host responses to brood parasites near the nest conform to these general ecological patterns as suggested by the present study.
